# Family-Based Discrimination, Non-disclosure of Sexual Orientation or Gender identity, and Initiation of Oral Pre-exposure Prophylaxis Among Adolescents

**DOI:** 10.1007/s10461-026-05075-9

**Published:** 2026-03-09

**Authors:** Gabriel Marinho, Beo Oliveira Leite, Diana Zeballos, Fabiane Soares, Nila Mara Smith Galvão, Leila Denise Amorim, Suelen Seixas, Dirceu Greco, Alexandre Grangeiro, Ines Dourado, Laio Magno

**Affiliations:** 1 Instituto de Saúde Coletiva, Universidade Federal da Bahia, Rua Basílio da Gama, s/n, Canela, Salvador, 40110-040 Bahia Brazil; 2 Departamento de Ciências da Terra, Universidade do Estado da Bahia, Rua Silveira Martins, 2555, Cabula, Salvador, 41150-000 Bahia Brasil; 3 Universidade Federal da Bahia, Instituto de Matemática e Estatística, Avenida Milton Santos, s/n, Ondina, Salvador, 40170-110 Bahia Brazil; 4 Departamento de Ciências da Vida, Universidade do Estado da Bahia, Rua Silveira Martins, 2555, Cabula, Salvador, 41150-000 Bahia Brazil; 5 Faculdade de Medicina, Universidade Federal de Minas Gerais, Avenida Professor Alfredo Balena, 190, Santa Efigênia, Belo Horizonte, 30130-100 Minas Gerais Brazil; 6 Faculdade de Medicina, Universidade de São Paulo, Avenida Dr. Arnaldo, 455, Cerqueira César, São Paulo, 01246-903 São Paulo Brazil; 7 Instituto Gonçalo Moniz, Fundação Oswaldo Cruz, Rua Waldemar Falcão, 121, Candeal, Salvador, 40296-710 Bahia Brazil

**Keywords:** Discrimination, Family, Pre-exposure prophylaxis, Men who have sex with men, Transgender women, Adolescents, Latent class analysis

## Abstract

This study analyzed the association between family-based discrimination and non-disclosure of sexual orientation or gender identity (SOGI) and the initiation of oral pre-exposure prophylaxis (PrEP) for HIV prevention among adolescent men who have sex with men (AMSM) and transgender women (ATGW). This analysis used baseline data from the PrEP1519 project. Latent class analysis (LCA) was applied to identify patterns of family-based discrimination and non-disclosure of SOGI using three categorical indicators. Descriptive, bivariate, and multivariate analyses were conducted, and logistic regression models estimated the adjusted odds ratios (aOR) for the association between latent classes and PrEP initiation. The majority of the 1,309 participants identified as AMSM (91.4%) were aged 18–19 years (74.9%), were Black and *Pardo* or mixed race (72.1%), had enrolled in high school (71.9%), resided in São Paulo (49.6%), and lived with parents or other family members (81.9%). Overall, 79.5% of patients initiated oral PrEP. LCA identified three distinct profiles: (i) low discrimination (67.6%, *n* = 885), (ii) high discrimination (22.4%, *n* = 293), and (iii) no disclosure within the family context (10.0%, *n* = 131). Multivariate analysis revealed non-disclosure of SOGI within the family was significantly associated with lower odds of initiating oral PrEP (aOR: 0.54; 95% CI: 0.33–0.78), whereas high levels of family discrimination were not significantly associated with oral PrEP initiation (aOR: 1.22; 95% CI: 0.84–1.76. Enhancing, structural interventions, stronger community support systems, and expanded access to alternative prevention options such as injectable PrEP may improve the initiation and adherence to HIV-prevention efforts among adolescents who experience family-related barriers to disclosure.

**Supplementary Information:**

The online version contains supplementary material available at 10.1007/s10461-026-05075-9.

## Resumen

Este estudio analizó la asociación entre la discriminación familiar y la no revelación de la orientación sexual o identidad de género (OSIG) y el inicio de la profilaxis preexposición oral (PrEP) para la prevención del VIH entre adolescentes hombres que tienen sexo con hombres (AHSH) y mujeres transgénero (AMT). Este análisis utilizó datos de la línea de base del proyecto PrEP1519. Se aplicó un análisis de clases latentes (LCA) para identificar patrones de discriminación familiar y la no revelación de la OSIG utilizando tres indicadores categóricos. Se realizaron análisis descriptivos, bivariados y multivariados, y se utilizaron modelos de regresión logística para estimar las odds ratio ajustadas (aOR) de la asociación entre las clases latentes y el inicio de la PrEP. La mayoría de los 1309 participantes se identificaron como AHSH (91,4 %), tenían entre 18 y 19 años (74,9 %), eran negros y pardos o mestizos (72,1 %), estaban matriculados en la escuela secundaria (71,9 %), residían en São Paulo (49,6 %) y vivían con sus padres u otros familiares (81,9 %). En general, el 79,5 % de los participantes iniciaron la PrEP oral. El LCA identificó tres perfiles distintos: i) baja discriminación (67,6 %, n = 885), ii) alta discriminación (22,4 %, n = 293) y iii) no revelación en el contexto familiar (10,0 %, n = 131). El análisis multivariante mostró que la no revelación de la OSIG a la familia esta asociada significativamente con menores probabilidades de iniciar la PrEP oral (aOR: 0,51; IC del 95 %: 0,33–0,78), mientras que los niveles altos de discriminación familiar no están asociados con el inicio de la PrEP oral (aOR: 1,22; 95 %: 0,84–1,76). El fortalecimiento de las intervenciones estructurales, sistemas de apoyo comunitario más sólidos y la ampliación del acceso a opciones alternativas de prevención, como la PrEP inyectable, pueden mejorar el inicio y la adherencia a los esfuerzos de prevención del VIH entre adolescentes que enfrentan barreras familiares para la revelación de la OSIG.

## Introduction

The global response to Human immunodeficiency virus (HIV) continues to advance toward the goal of ending acquired immunodeficiency syndrome (AIDS) as a public health threat by 2030. However, the decline in the number of new HIV infections remains insufficient to meet this target. The epidemic continues to disproportionately affect key populations, such as sex workers, people who inject drugs, men who have sex with men (MSM), and transgender women (TGW). Collectively, these groups will account for 55% of the newly diagnosed global HIV infections in 2022 [1]. Adolescents and young people aged 15–19 years within these key populations have also been significantly impacted by the epidemic, in part due to pervasive HIV-related stigma and limited social support, which are associated with poorer mental health outcomes, such as depression and suicidal thoughts [1–3], and with reduced engagement in HIV prevention and care, including delayed testing and lower use of preventive strategies [1, 4–6].

Although the overall incidence of HIV has been declining globally, Brazilian data show that the number of cases among young and adolescent men who have sex with men (AMSM) and adolescent transgender women (ATGW) has continued to increase over the last few decades [7]. This highlights the need for more targeted and effective prevention strategies for adolescents belonging to these key populations, who may be at an increased risk of sexually transmitted infections (STIs) due to factors such as the onset of sexual activity, engagement in risky behaviors, and a common sense of invulnerability [8].

Brazil’s Ministry of Health published the 2022 update of the Clinical Protocol and Therapeutic Guidelines for HIV Pre-Exposure Prophylaxis, lowering the minimum eligible age to 15 years and thus expanding pre-exposure prophylaxis (PrEP) access for adolescents who are sexually active, weighing at least 35 kg, and presenting an increased risk of HIV infection according to their sexual behavior and vulnerability context [9]. PrEP, which involves the use of antiretroviral medications administered generally orally or by injection, is a highly effective strategy for the prevention of HIV infection [10–14]. In Brazil, only oral PrEP is available in the Brazilian Public Health System (*Sistema Único de Saúde*) [9]. Studies examining MSM and TGW populations have demonstrated that PrEP is one of the most effective HIV prevention strategies when used as prescribed. Its effectiveness is closely linked to adherence and consistent use, which is essential for achieving the desired protective effect [11, 12, 15–17].

Unfortunately, oral PrEP uptake among adolescents and young adults aged 15–24 years in Brazil remains low, posing a major challenge to curbing the HIV epidemic [7]. Furthermore, adolescents still face significant barriers in accessing PrEP [18, 19]. In the United States, a study evaluating AMSM and ATGW conducted by Macapagal et al. [20] identified key obstacles to oral PrEP initiation, including concerns about side effects, limited knowledge of access routes, financial constraints, low-risk perception, stigma stemming from both HIV and sexual orientation or gender identity (SOGI), and discrimination. Similarly, in a Brazilian study that included MSM and TGW, Batista et al. [21] reported family-related barriers such as poor communication about sexuality, fear of familial stigma, and lack of parental support as key obstacles.

Families play a key role in emotional regulation and decision-making, especially during adolescence, a period marked by heightened vulnerability and change [22–24]. In a study with Black MSM aged 16–24, Boyd et al. [25] found that family support and positive attitudes were linked to greater perceived benefits of PrEP and increased acceptance. In contrast, Moskowitz et al. [26] reported that among MSM aged 13–18 years, fear of parental reactions and lack of openness about sexuality were associated with low interest in PrEP. Additionally, stigma and discrimination related to HIV/AIDS and PrEP remain significant barriers to uptake among adult MSM and TGW [27–29], as well as among AMSM and ATGW [30, 31].

Given the crucial role of family in adolescent development and health, it is essential to examine how family-based discrimination and openness to SOGI influence decision-making related to HIV prevention. This study examined the association between family-based SOGI discrimination, non-disclosure, and oral PrEP initiation among AMSM and ATGW. We hypothesized that family-based SOGI discrimination and non-disclosure would be negatively associated with oral PrEP initiation in these populations.

## Methods

### Study Design, Setting, and Population

This cross-sectional study used baseline data from the PrEP15-19 cohort, which aimed to evaluate the effectiveness of oral PrEP among AMSM and ATGW individuals at risk of HIV infection. The study was conducted between 2019 and 2023 in three Brazilian capital cities: Salvador, São Paulo, and Belo Horizonte.

The inclusion criteria for this study followed the general criteria of the PrEP15-19 project: (i) age between 15 and 19 years; (ii) living, working, or studying in Salvador, Belo Horizonte, or São Paulo; (iii) self-identifying as MSM or TGW; and (iv) having had at least one intercourse with another cisgender man or transgender woman in the past 12 months. Exclusion criteria included being under the influence of drugs at the time of the interview to a degree that compromised participation or assessment, and receiving a confirmed HIV-positive test result on the rapid test conducted during cohort screening.

Adolescents who met the general eligibility criteria were offered counselling on available HIV prevention options. Those who did not initiate PrEP were referred to alternative combination prevention strategies that did not involve PrEP, including consistent condom use, routine HIV testing, and sexual health counselling focused on risk reduction. Adolescents who expressed interest in initiating PrEP subsequently underwent additional clinical assessments to determine PrEP eligibility according to the criteria described by Dourado et al. [32]. Prior exposure PrEP was not considered an exclusion criterion.

### Recruitment and Data Collection

From February 2019 to December 2023, participants were recruited through the activities of peer educators for young people in public venues and institutions, digital platforms (Instagram, Facebook, WhatsApp, TikTok, YouTube, Spotify), and so-called “hook-up” or dating apps (Grindr, Hornet, Tinder, and Badoo). These efforts were also supported by drag queens and digital influencers [33].

Data were collected by regularly interviewing participants using structured questionnaires and a digital system for enrollment. The questionnaires were designed to assess different functions and covered domains such as demographic and socioeconomic characteristics, use of HIV prevention strategies, awareness and acceptance of PrEP, clinical history of STIs, and individual experience with discrimination and violence in different contexts. The answers provided by the participants were recorded on a dedicated electronic platform for the study by qualified professionals at each participating site. Further details on the data collection process are provided by Dourado et al. [32].

### Study Variables

To measure the latent exposure variables, three items from the socio-behavioral questionnaire were considered potential indicators for the LCA analysis (Box 1). Only variables that specifically captured family-based SOGI-related discrimination were included in the LCA. The answers “I do not know” and “I do not want to answer” were considered missing data and were excluded from the analysis, allowing each indicator to be treated as a variable with two or three categories in the analysis.

The first variable, family exclusion, refers to experiences of exclusion in the family environment owing to the affective–sexual life of the adolescent, that is, their emotional, romantic, and sexual relationships and the expression of their SOGI. The response categories for this variable were dichotomized between “Never” (0) and “At least once” (1). This indicator was chosen because it reflects the dynamics of acceptance, support, and stigma within the nuclear family, indicating isolation or rejection in the environment, and may be related to SOGI discrimination and non-disclosure. The second and third categorical variables, mother’s and father’s approval, refer to the approval of the adolescent’s sexuality (i.e., SOGI) by members of the nuclear family. Responses to these indicators were categorized as “Approves” (0), “Disapproves” (1), and “Unaware” (2). The last category was based on information from the existing literature on adolescents’ fear of possible discriminatory reactions by family members regarding their SOGI.

The socio-behavioral and sociodemographic variables, in addition to their respective response categories, used for the categorization of the AMSM and ATGW included in the PrEP15-19 cohort are described in Box 1. “PrEP initiation” within the PrEP15-19 study was defined as the outcome variable, and responses were based on whether the participant received (1) or did not receive (0) oral PrEP upon enrolling in the study or within 30 days of enrollment. The response was considered “yes” if oral PrEP was dispensed to the participant within 30 days of entering the study, and “no” otherwise.

Box 1. Sociodemographic and Socio-behavioral Variables Used for the Categorization of the Adolescent Men Who Have Sex with Men (AMSM) and Transgender Women (ATGW) Included in the PrEP15-19 Cohort

**Table:** 

Variables	Categories
Sociodemographic data	
Age	1. <18 years
	2. ≥18 years
Study population	(1) AMSM; (2) ATGW
Race/Color	(1) White; (2) Black and *Pardo*
Education	(1) Middle school; (2) High school; (3) Higher education
Housing situation	(1) Living with parents or relatives; (2) Living with partners or friends; (3) Living alone/no fixed address.
Employment	(1) Not employed; (2) Employed
Study site	(1) Salvador; (2) São Paulo; (3) Belo Horizonte
Health insurance	(1) Does not have insurance; (2) Has insurance
Socio-behavioral data	
Engaged in commercial sex work	(1) No; (2) Yes
Ever exchanged sex for money and/or favors	(1) Never; (2) At least once
Experienced SOGI-related aggression	(1) Never; (2) At least once
Tested for HIV before the study	(1) No; (2) Yes
Has used PEP	(1) No; (2) Yes
Outcome	
Started PrEP	(1) No; (2) Yes
Prior PrEP use	(1) No; (2) Yes
Items selected to LCA analysis	
Have you been excluded or marginalized in your family environment?	(1) Never; (2) At least once
How do the people you consider family deal with the fact that you have sex with men? – Mother	(1) Approve; (2) Disapprove; (3) Unaware
How do the people you consider family deal with the fact that you have sex with men? – Father	(1) Approve; (2) Disapprove; (3) Unaware

*LCA* latent class analysis

### Data Analysis

Latent class analysis (LCA) was used to construct the exposure variable “family-based SOGI discrimination and non-disclosure.” LCA is a statistical analysis procedure that seeks to empirically identify latent and distinct population subgroups through probability based on a set of expressed characteristics [34]. Weller et al. reported that the subgroups identified in LCA, called latent classes or groups, were detected based on participants’ responses to the categorical indicators assessed in the study [35]. Hence, LCA is used to identify response patterns to variables that describe underlying subgroups or categories that are not directly observable in the population. This means that LCA may explain the similarities between response patterns that cannot be directly observed in the collated data [35–38].

LCA was used to derive a latent construct representing a comprehensive multidimensional family-based SOGI stigma experience that could not be adequately captured by a single observed variable. Given that disclosure and discrimination are interrelated, context-dependent phenomena and can manifest in distinct but overlapping patterns, LCA provides a framework for empirically identifying underlying subgroups that reflect the combined structure of these experiences.

Two criteria were used to select indicators for this study. First, we ensured that the proportion of responses to the items was > 5%, to avoid possible issues when estimating the LCA model. Thereafter, we assessed the relationship between each indicator and the latent variable by comparing the conditional probabilities for each item and latent class. Considering the information reported by Collins et al. [38], we considered differences of < 25% for the same item in all classes indicative of a weak relationship or independence between the indicator and the “family-based SOGI discrimination and non-disclosure” latent variable.

Bayesian and Akaike information criteria were used as the selection criteria for the final LCA model, as both support the process of analysis by facilitating comparisons in terms of balancing model fit and parsimony, with lower values indicating a better fit of the model [38–40]. Moreover, the Lo–Mendell–Rubin likelihood ratio test was employed to evaluate whether there were statistically significant differences between the competing latent class models [41].

LCA was performed to compose the latent exposure variables using indicators from the socio-behavioral items in the questionnaire. The analysis was performed using Mplus v.8.10 (Muthén & Muthén, Los Angeles, CA, United States) software. After classifying the participants according to the highest probability of belonging, the categorical variable that represented the latent classes was entered into the database for external analyses (descriptive, bivariate, and multivariate) using the corresponding tools for the functions in the Stata v.15.0 (StataCorp LLC, College Station, Texas, United States) statistical software. This classification procedure regarding the latent variable, followed by its use in external analyses, is called the classify–analyze approach [42].

Bivariate analyses using chi-square tests were conducted to assess the associations between each covariate and both the exposure and outcome variables.

Potentially confounding sociodemographic and behavioral covariates used in the multivariate analysis were included in the final logistic model based on their theoretical relevance, association with the exposure variable, or outcome. The significance level was set at 10% (*p* < 0.10). Covariates whose removal resulted in a distortion in the main association measure closer to 5% (i.e., study site, health insurance status, and engagement in commercial sex work) were also included. In addition, 95% confidence intervals (CIs) were estimated for odds ratios (ORs). Bivariate and multivariate analyses and chi-square tests were conducted using STATA v. 15.0.

### Ethical Considerations

The study was approved by the World Health Organization (WHO) Research Ethics Committee (protocol ID: Fiotec-PrEP adolescent study), the Brazilian Research Ethics Committee, and the Ethics Committee for each city where the research coordination centers were located: Federal University of Bahia (#3.224.384), Federal University of Minas Gerais (#2.027.889), and University of São Paulo (#3.082.360). This study complied with Resolutions 466/2012 and 510/2016 of the Brazilian National Health Council, which established the requirement that all research involving humans be submitted to a Research Ethics Committee for approval.

Regarding adolescent participation in the study, legal decisions regarding the waiver of parental consent at each research site differed. A collective waiver was awarded to São Paulo. In Salvador, a waiver was granted only to adolescents in vulnerable or broken family bond situations. A collective waiver that required parental consent from all individuals under 18 years of age was denied in Belo Horizonte. Further information on participation waivers can be found in Zucchi et al. [43]. The right and autonomy of the participant to withdraw consent at any time were respected, ensuring their voluntary participation in the study. In addition, participants had the right to refuse to answer questions they found uncomfortable for any reason.

## Results

### Sociodemographic and Behavioral Profile of the Study Participants

A total of 1,309 participants were included, of whom 91.4% and 8.6% identified as MSM and TGW, respectively. Most of the participants (72.1%) identified as Black or *Pardo* (i.e., mixed-race people, according to the Brazilian Institute of Geography and Statistics [*Instituto Brasileiro de Geografia e Estatística* {IBGE}] [44] classification for race), attended high school (71.9%), and lived in São Paulo (49.6%). Most lived with their parents or other family members (81.9%), whereas 8.4% lived alone or had no fixed address. Regarding age, 74.9% of the participants were in the 18–19 years age group. More than half were unemployed (57.6%), and most lacked health insurance coverage (76.4%). Only 39 (2.97%) participants reported having used PrEP before study enrollment, of whom 37 were initiated in the PrEP15-19 study. PrEP was initiated within 30 days of the initial consultation by 79.5% of the participants (Table 1).

**Table 1:** Characteristics of adolescent men who have sex with men (AMSM) and transgender women (ATGW) who participated in the PrEP15-19 project: Brazil, 2019–2023 (*N* = 1,309)

Variable	*N*	%
Study site		
Salvador	402	30.7
São Paulo	649	49.6
Belo Horizonte	258	19.7
Study population		
MSM	1196	91.4
TGW	113	8.6
Race		
White	354	27.9
Black and *Pardo*	913	72.1
Education		
Middle school	90	6.9
High school	940	71.9
Higher education	277	21.2
Age group		
15–17 years	329	25.1
18–19 years	980	74.9
Family-based SOGI discrimination and non-disclosure		
Low family discrimination	885	67.6
High family discrimination	293	22.4
Family members are unaware of SOGI	131	10.0
Employment		
Working without income or not working	754	57.6
Working with income	555	42.4
Housing situation		
Living with parents or other family members	1072	81.9
Living with partners or friends	127	9.7
Living alone/No fixed address	104	7.9
Health insurance		
No	986	76.4
Yes	304	23.6
Commercial sex work		
No	1180	94.3
Yes	71	5.7
Ever exchanged sex for money and/or favors		
Never	1112	85.3
At least once	192	14.7
Experienced SOGI-related aggression		
Never	1224	93.7
At least once	82	6.3
Tested for HIV before the study		
No	572	43.7
Yes	737	56.3
Used PEP		
No	1168	89.2
Yes	141	10.8
Started PrEP		
No	268	20.5
Yes	1041	79.5
Prior PrEP use		
No	1270	97.0
Yes	39	2.97

Regarding socio-behavioral characteristics, a small proportion of the participants reported engaging in commercial sex work (5.7%) and 14.7% reported having sex in exchange for money and/or favors at least once. Additionally, 6.3% of the participants reported experiencing SOGI-related aggression. Regarding the prevention of HIV infection, 10.8% of the participants reported that they had used PEP, and 56.3% reported that they were tested for HIV before enrolling in the study (Table 1).

Almost half of the participants (45.1%) had experienced SOGI-related exclusion or marginalization in their families. In addition, approximately 15% of participants reported that their mothers disapproved of their SOGI. However, a slightly higher proportion of participants (22.4%) reported that their fathers disapproved of their SOGI. The proportion of participants whose mothers approved of the SOGI was higher (74.4%) than that of participants whose fathers expressed approval (52.1%). Conversely, the proportion of participants whose mothers were unaware of their SOGI was lower than the proportion of participants whose fathers were unaware (10.6% and 25.5%, respectively) (Table 2).

**Table 2:** Characterization of latent patterns of family-based discrimination and non-disclosure among AHSH and ATGW using latent class analysis: Brazil, 2019–2023 (*N* = 1,309)

		Patterns of family-based discrimination and non-disclosure of SOGI		
		Latent class prevalences (%)		
	Frequency in the general study population (%)	Low family discrimination (*n* = 885; 67.61%)	High family discrimination (*n* = 293; 22.38%)	Family members unaware (*n* = 131; 10.01%)
Indicators		Conditional probabilities (%)		
Were you excluded or marginalized in your family environment				
Never	54.9	**69.0**	**24.6**	**66.3**
At least once	45.1	**31.0**	**75.4**	**33.7**
How do the people you consider your family deal with the fact that you have sex with men? - Mother				
Approves	74.4	**98.7**	**53.7**	–
Disapproves	15.0	**0.6**	**46.0**	**4.6**
Unaware	10.6	**0.7**	**0.3**	**95.4**
How do the people you consider your family deal with the fact that you have sex with men? - Father				
Approves	52.1	**70.6**	**36.8**	–
Disapproves	22.4	**11.1**	**51.6**	**0.7**
Unaware	25.5	**18.4**	**11.6**	**99.3**

Values in bold indicate probabilities that are at least 25% higher or 25% lower than those observed in the general study population

*AMSM* adolescent men who have sex with men, *ATGW* adolescent transgender women

### Identification of Family-Based Discrimination and Non-disclosure Patterns

Two sequential LCA models were compared to evaluate the latent variable “Family-based SOGI discrimination and non-disclosure” (Table 2). The number of classes in the models ranged from two to three and were compared based on the criteria described in the Methods section. The Lo–Mendell–Rubin likelihood ratio test strongly favored the three-class solution over the competing models (LRT = 141.633, *p* > 0.05). Statistical criteria (Table 3) and factors such as class interpretability and model identifiability rendered the three-class LCA model the most suitable. This model had a good classification accuracy, which was corroborated by the average latent class posterior probabilities (ALCPP > 0.80). Table 2 presents the parameter estimates of the final latent class model. These include the following: (i) the frequency of indicators in the general study population, (ii) class prevalence, and (iii) conditional response probabilities for each indicator within each class, which describe how likely each response category distributed among individuals belonging to that subgroup.

**Table 3:** Fit statistics and classification coefficients for the latent class analysis models

Parameter	2 classes	3 classes
AIC	25,545.396	25,514.944
BIC	25,617.874	25,523.944
BIC-sample-size-adjusted	25,573.403	25,456.955
Entropy	0.834	0.688
ALCPP	0.947	0.832

*AIC* Akaike information criterion, *BIC* Bayesian information criterion, *ALCPP* average latent class posterior probability

The low family-based discrimination class comprised 67.6% of the individuals and was characterized by minimal parental disapproval (e.g., mother: 0.6%; father: 11.1%). The high family-based discrimination class included 22.4% of participants and showed substantial maternal (46%) and paternal (51.6%) disapproval, along with high levels of family-based SOGI discrimination (75.4%). The unawareness of family members class represented 10.0% of individuals and was associated with low parental disapproval (mother: 4.6%; father: 0.7%) but a very high likelihood that parents were unaware of the SOGI of the individual (mother: 95.4%; father: 99.3%).

Notably, only the high-discrimination class showed parental disapproval rates exceeding 45%, highlighting its markedly elevated level of SOGI-related discrimination compared with the low-discrimination class (Table 2).

### Final Multivariable Model Results

The bivariate analysis is described in Supplementary Material 1. The final model, based on the results of the multivariate analysis, was adjusted for the following covariates: housing situation, history of commercial sex work, age group, study site, and health insurance. The analysis indicated that SOGI non-disclosure within the family context was associated with a lower chance of initiating PrEP (aOR: 0.51; 95% CI: 0.33–0.78) compared to a low level of discrimination in the family. Conversely, a high level of family discrimination was not significantly associated with PrEP initiation (aOR: 1.22; 95% CI: 0.84–1.76) (Table 4).

**Table 4:** Association between family-based SOGI discrimination and non-disclosure and PrEP initiation use among AMSM and ATGW who participated in the PrEP15-19 project: Brazil, 2019–2023 (*N* = 1309)

Variables		Final model¹
	Crude OR (95% CI)	aOR¹ (95% CI)
Family-based SOGI discrimination		
Low family discrimination	-	-
High family discrimination	1.21 (0.85–1.71)	1.22 (0.84–1.76)
Family members are unaware	0.54 (0.36–0.82)	0.51 (0.33–0.78)

Model adjusted for: study site, age group, housing situation, engagement in commercial sex work, and health insurance status

*AMSM* adolescent men who have sex with men, *ATGW* adolescent transgender women, *SOGI* sexual orientation or gender identity

## Discussion

The first hypothesis that family-based SOGI discrimination is associated with oral PrEP initiation was not supported by our data. In contrast, the second hypothesis, postulating a negative association between family-based SOGI nondisclosure and the initiation of oral PrEP among these populations, was confirmed.

Participants who did not reveal their SOGI to their families may have anticipated discrimination, resulting in more implicit forms of tension in their relationships, which may have discouraged any interest in initiating PrEP. A lack of family support can limit the autonomy of an adolescent in decision-making about their sexual health, such as initiating PrEP [45–47]. In addition, family engagement and support contribute to improved health outcomes [48, 49] and help reduce the stigma associated with PrEP, thereby promoting its acceptance and adoption [25, 50, 51]. Family has been identified as a key factor that can either facilitate or hinder PrEP use, with several studies highlighting the link between stigma emerging as a central mechanism linking family dynamics and decreased PrEP uptake [52, 53].

The study findings are consistent with evidence from previous studies conducted among adolescents and young adults in the United States, which showed that fear or non-disclosure of SOGI constitutes an obstacle to accessing PrEP [26, 29, 54, 55], often leading adolescents to avoid discussions related to these topics. Another study from the United States by Boyd et al. [25] found that young people exposed to discrimination or family rejection were more hesitant to seek information about PrEP and were less likely to adhere to its use. Additional studies have indicated that the expectation of gender identity-related rejection among TGW is associated with sexual behaviors that increase the risk of HIV infection [51, 56]. Collectively, these findings highlight the potentially harmful effects of family discrimination on health. Conversely, a supportive family environment facilitates a sense of comfort for adolescents when discussing their sexual health needs and PrEP usage [51, 55, 57].

Non-disclosure of SOGI to one’s family may be both a consequence of anticipating stigma and discrimination [49, 51, 58, 59] and a protective strategy against these factors [58, 60]. However, this can hinder initiation of PrEP [59, 61]. The iPrEx study, a qualitative study including 32 MSM participants conducted in Thailand, indicated that non-disclosure of SOGI to family members hindered participation in the study and led participants to hide medications to avoid questioning [59].

Fear of family surveillance of sexuality and restriction of autonomy can hinder the use of PrEP by adolescents at home [45, 51, 58]. Reports of PrEP users hiding medications from their families have been widely published [46, 48, 51, 59, 62]. Moreover, fear of family judgment is a significant obstacle to starting and continuing to use PrEP [59, 61]. In Kenya, participants in a qualitative study associated PrEP stigma with the fear of family members seeing them take the pill [46]. Furthermore, PrEP may be considered by family members as indicative of “promiscuous” or “immoral” behavior among young people [45, 46]. In this context, a lack of family support can intensify logistical barriers for AMSM and ATGW as individuals in this age group are more likely to rely on family networks for financial and emotional support and may have limited autonomy or skills to advocate for their own health needs. Concerns about confidentiality, such as the need to conceal PrEP pills or clinic appointments from family members, can create stressful situations that adolescents may feel less equipped to manage than older individuals [35]. Beyond the challenges commonly experienced by older individuals, AMSM and ATGW also face additional obstacles in navigating complex health care systems and accessing confidential HIV prevention services, which together contribute to lower uptake and adherence to daily oral PrEP in these groups [29, 34].

Some studies suggest that secrecy functions as a means of preserving autonomy in restrictive family environments. Qualitative research conducted by Moskowitz et al. in the United States, which included 491 AMSM aged 13–18 years, indicated that adolescents take PrEP secretly to maintain autonomy by avoiding judgment and parental control [26, 54]. An evaluation of the combined cohorts of the two studies [55, 57] showed that more than 80% of the participants stated that they would use PrEP if they could do so without their parents’ knowledge. However, the results of the present study diverge from this pattern, instead reinforcing the essential role of family support and knowledge of SOGI in the decision to initiate PrEP use, which is also supported by findings from several studies [25, 50, 51, 55, 63, 64].

In some contexts, family members directly support PrEP use by reminding users to take their medication or count the remaining pills [64]. Furthermore, family awareness of PrEP and its properties could increase the rate of PrEP initiation in Kenya and South Africa [65]. Similarly, a qualitative study conducted by Flores et al. (2020), which included 222 AMSM in the United States, showed that being comfortable communicating with parents about sex was positively associated with PrEP use [63]. Previous studies have revealed that greater social support, including family support, not only increases HIV testing and PrEP use [26, 63, 66] but also reduces the stigma associated with the medication [25]. Furthermore, studies conducted in Africa and North America have shown that family and parental relationships are the primary sources of support for PrEP use [49, 51, 55, 64]. Thus, family dynamics can function as both barriers and facilitators in decision-making processes related to adolescents ‘sexual health.

The association between SOGI non-disclosure and lower PrEP initiation highlights some social implications. The requirement of daily oral PrEP exposes medication use within the household, increasing the possibility of inadvertent disclosure through parental surveillance. This fear of being “outed” at home may be a barrier to PrEP uptake among adolescents who conceal their SOGI from family members. Therefore, an injectable PrEP approach is a viable alternative. Injectable PrEP requires administration only once every two months (cabotegravir) or every six months (lenacapavir), and can directly address the barrier of daily medication visibility and the associated possibility of family detection, providing greater privacy and reducing the requirement for justifications for its use. This is perceived as an approach that affords increased privacy and autonomy, particularly among individuals who live with family members resistant or intolerant to PrEP, homosexuality, transsexuality, and gender-nonconforming expressions, as consistently highlighted in previous studies [67–69].

In contrast to the findings related to non-disclosure, higher family discrimination showed no statistically significant association with PrEP initiation compared with lower family discrimination. This finding contradicts our initial hypothesis and runs counter to the existing evidence that highlights high family discrimination as an important obstacle to PrEP initiation [25, 51, 70]. Nevertheless, the point estimate suggested an elevated likelihood of PrEP treatment initiation in the high family-based discrimination group. One possible explanation is that within family contexts where SOGI is already known, disclosure itself may carry greater social and emotional weight than experiences of discrimination. In this context, adolescents may feel less constrained by concerns regarding concealment or shame related to PrEP use. Accordingly, the study hypothesized that adolescents who experienced family-based SOGI discrimination may be less inhibited from initiating PrEP than those whose SOGI remain undisclosed within the family. Further qualitative studies are warranted to explore these dynamics in greater depth and better understand how family dynamics, including disclosure, discrimination, and support, intersect to shape decision-making by adolescents regarding PrEP initiation.

Other studies have reported counterintuitive associations between discrimination experience and PrEP-related outcomes. For instance, a Brazilian study within the PrEP15-19 cohort conducted by Soares et al. [71], which included 908 adolescents aged 15–19 years, revealed a significant association between PrEP initiation and previous experience of SOGI discrimination. A study by Quinn et al., which included a sample of 283 adolescent and young adult MSM, reported that, although major discriminatory events were not associated with PrEP outcomes, more frequent day-to-day experience with intersectional discrimination were positively associated with the likelihood of future PrEP use [72]. These findings are consistent with other studies that have documented complex and nonlinear relationships between discrimination-related processes and engagement in HIV prevention. For example, a higher degree of internalized racism has been associated with higher PrEP uptake [73]. Similarly, a higher degree of homophobia has also been observed among MSM who use PrEP than among those who do not [74]. Sexuality-related discrimination has also been positively associated with PrEP use in other contexts [75]. Taken together, this body of evidence highlights the multifaceted nature of discrimination and suggests the need to move beyond linear assumptions about its relationship with HIV-prevention behaviors [72].

Support for the multifaceted nature of discrimination pattern lies in the growing body of research emphasizing the role of resilience in shaping health-related behaviors among sexual and gender minority populations [76, 77]. Rather than being a fixed trait, resilience is understood as a dynamic process through which individuals adapt to adverse social conditions and mobilize available resources [78, 79]. In the context of family-based discrimination, positive adaptations may occur through protective internal assets at the individual level as well as through external resources such as supportive social environments that promote favorable health outcomes [80].

These resources include social support [81–83] and engagement with affirming sexual and gender minority communities [84, 85]. Experience with discrimination may activate coping processes and foster adaptive strategies, such as seeking social support and community belonging [72, 86–88]. In line with this interpretation, it is plausible that AMSM and ATGW who report high levels of family discrimination compensate for the lack of familial support through external support networks, such as friends, sexual and gender minority peers, affirming community spaces, and specialized sexual health services [16, 89]. These connections may, in turn, positively impact PrEP outcomes by increasing exposure to affirming PrEP messages, enhancing knowledge of peers on PrEP use, and improving access to prevention methods [85, 90].

Qualitative studies among MSM [90] and sexual and gender minority youth [91] further illustrate how individuals navigate discriminatory environments by mobilizing internal and external resources. From this perspective, PrEP use may also function as a symbolic form of self-protection against emotional and cultural barriers present in violent or discriminatory social environments, such as a highly discriminatory family. Additionally, some individuals may respond to the lack of family support through intensified self-care and risk perception, both of which are associated with an active search for HIV prevention methods [54, 92, 93].

Considering the critical role of family members, especially parents, in HIV prevention, greater effort is required to promote discussions on SOGI between adolescents and their families [94]. The unawareness and invisibility of SOGI manifested by family members, reinforces the emotional and cultural barriers to the adoption of preventive practices [95, 96]. Increasing parental awareness of PrEP and promoting dialogue regarding a child’s sexuality can not only facilitate PrEP initiation among AMSM and ATGW [26] but may also reduce the associated stigma [25]. Family-based interventions and research on PrEP use and the reduction of associated stigma should be explored to improve the PrEP care continuum [55, 96]. In addition, clinical discussions can be established as strategies to circumvent anticipated stigma, which can lead to the non-disclosure of SOGI to family members [61]. Furthermore, preventive strategies adapted to user needs can encourage PrEP use [97]. Previous studies have highlighted PrEP as a beneficial strategy when individuals are confronted with the possibility of family discrimination [54, 65, 98, 99].

This study has some limitations that should be acknowledged. First, the use of interview instruments that, despite providing relevant data, were not originally developed to measure discrimination and/or support in family contexts presented some challenges in capturing the subjective and contextual nuances of discrimination and support in the family. Second, differentiating between the specific sources of family discrimination (parents, siblings, or other members) was not possible in some cases. This limited the inclusion of indicators in the composition of the latent exposure variable, which was limited to three indicators, was considerably closer to indicating parental bias, and did not reflect the full diversity of family composition.

Additionally, because the latent construct was designed to capture family-based SOGI-related discrimination using indicators specifically referring to same-sex sexual behavior (e.g., parental approval of sexual relations with men), the model may not fully represent the experience of ATGW. For cisgender AMSM, discrimination expressed by family members was more directly linked to sexual behavior, whereas for ATGW, discrimination was often tied to gender identity and sexuality. The available indicators did not specifically measure stigma related to gender identity within the family.

Although the inclusion of both the AMSM and ATGW is a strength of the study, the analytical strategy adopted and the scope of the discussion did not fully differentiate between these populations. This approach may obscure meaningful differences in how family dynamics, stigma, and support operate across SOGI. Family-based challenges related to PrEP-use are qualitatively distinct for AMSM and ATGW, given the centrality of gender identity-related stigma in the latter group. Future studies could benefit from a disaggregated analysis or from qualitative and mixed-method approaches that are capable of capturing unique family related challenges regarding PrEP.

Another limitation concerns the LCA. Although it has been consolidated since 1950 [100], its computational implementation is much more recent, and its application to empirical data is still being explored. One of its main limitations is the identification of subgroups based on individual probabilities, which can compromise classification accuracy [36, 37]. Fourth, “naming fallacy” represents a methodological limitation of this study because the complexity of naming latent classes can lead to misinterpretations of the analyzed phenomena [35]. Specifically, the method used to classify family discrimination in this study did not completely capture the complexity of family relationships or support dynamics in this context. The classification of “high family discrimination” may encompass different realities, from complete rejection to situations of conflict, although the presence of other compensatory factors may weaken the direct association with PrEP initiation observed in the multivariate analysis.

In addition, although several criteria were used to select the optimal number of classes, such as the AIC, BIC, and likelihood-ratio tests, the low entropy value in this study may represent an additional limitation, as it is considered associated with reasonably poorer classification [36]. Lower entropy values indicate greater classification uncertainty and may contribute to underestimation of the effects in subsequent analyses. Furthermore, a classification analysis strategy was used in this study; that is, a latent class indicator variable was used in the external analyses by importing the variable from Mplus into Stata. Inherent measurement errors deriving from this method can affect the accuracy of the statistical estimates and results, which were not considered when evaluating the association between the construct and PrEP initiation. However, these results can still be validated using alternative methods, such as the Lanza, Tan, and Bray approach [42].

A possible bias in this study lies in the participant selection process, particularly due to differences in consent procedures across the study sites. The parental consent required for the inclusion of adolescents in Belo Horizonte’s research center may have systematically excluded youths whose legal guardians were unaware of or did not support their SOGI, leading to an underrepresentation of individuals in family contexts characterized by high secrecy or rejection. As a result, the sample may have been biased toward adolescents with greater family approval, compromising the generalizability of the findings. However, two of the three sites (Salvador and São Paulo) allowed for assent without mandatory parental involvement, enabling the inclusion of participants from a broader range of family contexts, including those who had potentially experienced family-based SOGI discrimination. Moreover, factors such as the misclassification of latent variables or variability due to sample size may inflate or distort the magnitude of the effect. Thus, caution is warranted when interpreting these results and recognizing the possibility of biases inherent in the study design and analysis.

## Conclusions

This study highlights the role of parental discrimination and non-disclosure of SOGI in shaping initiation of PrEP use. Compared with adolescents experiencing low levels of family-based SOGI discrimination, those who had not disclosed their SOGI to family members had lower odds of initiating oral PrEP. In contrast, a higher degree of family-based SOGI discrimination was not significantly associated with PrEP initiation. These findings suggest that SOGI non-disclosure within the family reflects anticipated discrimination. This assumption may stem from fear of parental conflict and negative reactions as well as from a perceived restriction of personal autonomy in adolescence. The findings of this study reinforce the critical role of family support and open communication in the prevention of HIV. In addition, the results encourage future research on discrimination and family dynamics in the AMSM and ATGW populations to facilitate the development of inclusive and effective public health policies. Furthermore, this study emphasized the need to adapt PrEP to specific populations based on an understanding of how families can influence decisions related to its use.

## Supplementary Information

Below is the link to the electronic supplementary material.

Supplementary Material 1
