# Supplementary material for: Family-Based Discrimination, Non-disclosure of Sexual Orientation or Gender identity, and Initiation of Oral Pre-exposure Prophylaxis Among Adolescents
Source: AIDS Behav. 2026 Mar 9;30(8):2488–502. doi: 10.1007/s10461-026-05075-9 (PMC13486071; doi:10.1007/s10461-026-05075-9)
Supplement: Supplementary file 1 [file 10461_2026_5075_MOESM1_ESM.pdf]

Table 5. Bivariate analysis of PrEP initiation and the sociodemographic and behavioral characteristics of adolescent AMSM and ATGW who participated in the PrEP1519 project. Brazil, 2019–2023.

Variables	PrEP initiation				p-value
	No		Yes		
	n	%	N	%	
**Study population**					0.649
MSM	243	90.67	953	91.55	
TGW	25	9.33	88	8.45	
**Age group**					0.830
15–17 years	66	24.63	263	25.26	
18–19 years	202	75.37	778	74.74	
**Study site**					0.000
Salvador	110	41.04	292	28.05	
São Paulo	107	39.93	542	52.07	
Belo Horizonte	51	19.03	207	19.88	
**Race/Color**					0.832
White	71	20.06	283	79.94	
Black and Brown	188	20.59	725	79.41	
**Education**					0.533
Middle school	22	8.24	68	6.54	
High school	186	69.66	754	72.50	
Higher education	59	22.10	218	20.96	
**Health insurance**					0.154
No	209	79.77	777	75.58	
Yes	53	20.23	251	24.42	
**Housing situation**					0.963
Living with parents or other family members	218	82.26	854	82.27	
Living with partners or friends	25	9.43	102	9.83	
Living alone/no fixed address	22	8.30	82	7.90	
**Employment**					0.141
Working without income or not working	165	61.57	589	56.58	
Working with income	103	38.43	452	43.42	
**Commercial sex work**					0.608
No	246	94.98	934	94.15	
Yes	13	5.02	58	5.85	
**Ever exchanged sex for money and/or favors**					0.221
Never	234	87.64	878	84.67	
At least once	33	12.36	159	15.33	

**Has been forced into having sexual intercourse**					0.079
Never	203	76.03	725	70.59	
At least once	64	23.97	302	29.41	
**Has experienced SOGI-related aggression**					0.425
No	254	76.03	970	70.59	
Yes	14	23.97	68	29.41	
**Tested for HIV before the study**					0.69
No	120	44.78	452	43.42	
Yes	148	55.22	589	56.58	
**Used PEP**					0.68
No	241	20.63	927	79.37	
Yes	27	19.15	114	80.85	
**Used a condom during first sexual intercourse**					0.742
No	141	89,93	566	89.05	
Yes	113	10.07	433	10.05	
**Patterns of family-based discrimination and non-disclosure**					0.003
Low family discrimination	177	66.04	708	68.01	
High family discrimination	50	18.66	243	23.34	
Family members are unaware	41	15.30	90	8.65	

*p-value estimated using the chi-square test

Table 6. Bivariate analysis of family-based SOGI discrimination and non-disclosure and the sociodemographic and behavioral characteristics of adolescent men who have sex with men (MSM) and transgender women (TGW) who participated in the PrEP1519 project. Brazil, 2019–2023.

Variables	Family-based SOGI discrimination and non-disclosure						p-value*
	Low family discrimination		High family discrimination		Family members unaware		
	n	%	n	%	n	%	
**Study population**							0.302
MSM	808	91.30	264	90.10	124	94.66	
TGW	77	8.70	29	9.90	7	5.34	
**Age group**							0.090
15–17 years	226	25.54	80	27.30	23	17.56	
18–19 years	659	74.46	213	72.70	108	82.44	
**Study site**							0.680
Salvador	274	30.96	85	29.01	43	30.71	
São Paulo	439	49.60	152	51.88	58	49.58	
Belo Horizonte	172	19.44	56	19.11	30	19.71	
**Race/Color**							0.479
White	231	27.08	88	30.77	35	27.34	
Black and Brown	622	72.92	198	69.23	93	72.66	
**Education**							0.520
Middle school	54	6.10	26	8.93	10	7.63	
High school	639	72.20	205	70.45	96	73.28	
Higher education	192	21.69	60	20.62	25	19.08	
**Health insurance**							0.989
No	670	76.48	217	76.14	99	76.43	
Yes	206	23.52	68	23.86	30	23.57	
**Housing situation**							0.033
Living with parents or other family members	729	82.65	227	78.28	151	88.55	
Living with partners or friends	86	9.75	36	12.76	6	3.05	
Living alone/no fixed address	67	7.60	30	8.97	17	8.40	
**Employment**							0.193
Working without income or not working	497	56.16	173	59.04	84	64.12	
Working with income	388	43.84	120	40.96	47	35.88	
**Commercial sex work**							0.504
No	806	94.82	261	93.55	113	92.62	
Yes	44	5.18	18	6.45	9	7.38	

**Ever exchanged sex for money and/or favors**							0.892
Never	755	85.60	247	84.59	110	84.62	
At least once	127	14.40	45	15.41	20	15.38	
**Has been forced into having sexual intercourse**							0.392
Never	628	71.53	212	74.13	88	67.69	
At least once	250	28.47	74	25.87	42	32.31	
**Has experienced SOGI-related aggression**							0.254
No	826	93.44	278	95.53	120	91.60	
Yes	58	6.56	13	4.47	11	8.40	
**Tested for HIV before the study**							0.628
No	386	43.62	124	42.32	62	47.33	
Yes	499	56.38	169	57.68	69	52.67	
**Used PEP**							0.504
No	794	89.72	256	87.37	118	90.08	
Yes	91	10.28	37	12.63	13	9.92	
**Used a condom during first sexual intercourse**							0.692
No	484	57.14	154	54.23	69	56.56	
Yes	363	42.86	130	45.77	53	43.44	
**PrEP initiation**							0.003
No	177	20.00	50	17.06	41	31.30	
Yes	708	80.00	243	82.94	90	68.70	

*p-value estimated using the chi-square test
